# Oral drugs for hypertensive urgencies: systematic review and meta-analysis

**DOI:** 10.1590/S1516-31802009000600009

**Published:** 2010-05-21

**Authors:** Luciana Mendes Souza, Rachel Riera, Humberto Saconato, Adriana Demathé, Álvaro Nagib Atallah

**Affiliations:** I MD. Postgraduate (MSc) student in the Discipline of Emergency Medicine and Evidence-Based Medicine, Universidade Federal de São Paulo - Escola Paulista de Medicina (Unifesp-EPM), São Paulo, Brazil.; II MD, MSc. Research assistant in the Brazilian Cochrane Center and physician in the Discipline of Emergency Medicine and Evidence-Based Medicine, Universidade Federal de São Paulo - Escola Paulista de Medicina (Unifesp-EPM), São Paulo, Brazil.; III MD, MSc, PhD. Assistant professor in the Department of Medicine, Universidade Federal do Rio Grande do Norte (UFRN), Natal, Rio Grande do Norte, Brazil.; IV MD, MSc. Postgraduate (PhD) student in the Department of Pathology and Propaedeutics, Universidade Estadual de São Paulo (Unesp), Araçatuba, São Paulo, Brazil.; V MD, MSc, PhD. Full professor and Head of the Discipline of Emergency Medicine and Evidence-Based Medicine of Universidade Federal de São Paulo - Escola Paulista de Medicina (Unifesp-EPM), and Director of the Brazil­ian Cochrane Center, São Paulo, Brazil.

**Keywords:** Hypertension, Review [Publication Type], Antihypertensive agents, Angiotensin-converting enzyme inhibitors, Calcium channel blockers, Hipertensão, Revisão [tipo de publicação], Anti-hipertensivos, Inibidores da enzima conversora da angiotensina, Bloqueadores dos canais de cálcio

## Abstract

**CONTEXT AND OBJECTIVE:**

Hypertensive urgencies are defined as severe elevations in blood pressure without evidence of acute or progressive target-organ damage. The need for treatment is considered urgent but allows for slow control using oral or sublingual drugs. If the increase in blood pressure is not associated with risk to life or acute target-organ damage, blood pressure control must be implemented slowly over 24 hours. For hypertensive urgencies, it is not known which class of antihypertensive drug provides the best results and there is controversy regarding when to use antihypertensive drugs and which ones to use in these situations. The aim of this review was to assess the effectiveness and safety of oral drugs for hypertensive urgencies.

**METHODS:**

This systematic review of the literature was developed at the Brazilian Cochrane Center, and in the Discipline of Emergency Medicine and Evidence-Based Medicine at the Universidade Federal de São Paulo - Escola Paulista de Medicina (Unifesp-EPM), in accordance with the methodology of the Cochrane Collaboration.

**RESULTS:**

Sixteen randomized clinical trials including 769 participants were selected. They showed that angiotensin-converting enzyme inhibitors had a superior effect in treating hypertensive urgencies, evaluated among 223 participants. The commonest adverse event for calcium channel blockers were headache (35/206), flushing (17/172) and palpitations (14/189). For angiotensin-converting enzyme inhibitors, the principal side effect was bad taste (25/38).

**CONCLUSIONS:**

There is important evidence in favor of the use of angiotensin-converting enzyme inhibitors for treating hypertensive urgencies, compared with calcium channel blockers, considering the better effectiveness and the lower frequency of adverse effects (like headache and flushing).

## RESUMO

**CONTEXTO E OBJETIVO:**

Urgências hipertensivas são definidas como elevações graves na pressão arterial sem evidência de danos agudos ou progressivos a órgãos-alvo. A necessidade de tratamento é considerada urgente, mas permite um controle gradual, utilizando-se drogas orais ou sublinguais. Se o aumento na pressão arterial não está associado a risco de vida ou danos a órgãos alvo, o controle pressórico deve ser feito lentamente durante 24 horas. Em relação às urgências hipertensivas, não é conhecida qual a classe de drogas anti-hipertensivas que promove os melhores resultados e há controvérsia em relação a quando e quais as drogas devem ser utilizadas nestas situações. O objetivo desta revisão foi avaliar a efetividade e a segurança de drogas orais para urgências hipertensivas.

**METODOS:**

Esta revisão sistemática da literatura foi desenvolvida no Centro Cochrane do Brasil, e na Disciplina de Medicina de Urgência e Medicina Baseada em Evidências da Universidade Federal de São Paulo - Escola Paulista de Medicina (Unifesp-EPM), de acordo com a metodologia da Colaboração Cochrane.

**RESULTADOS:**

Os 16 ensaios clínicos aleatórios selecionados incluíram 769 participantes e demonstraram um efeito superior dos inibidores da enzima conversora de angiotensina no tratamento da urgência hipertensiva, avaliada em 223 participantes. Os efeitos adversos mais frequentes para os bloqueadores de canal de cálcio foram cefaleia (35/206), rubor (17/172) e alterações do ritmo cardíaco (14/189); para os inibidores da enzima conversora de angiotensina, o efeito colateral mais frequente foi disgeusia (25/38).

**CONCLUSÕES:**

Há evidências importantes a favor do uso de inibidores da enzima conversora da angiotensina para o tratamento de urgências hipertensivas, quando comparados aos bloqueadores dos canais de cálcio, devido a maior efetividade e à menor frequência de efeitos adversos, como cefaléia e rubor facial.

## INTRODUCTION

Hypertensive crises have been divided into two categories: hypertensive urgencies and hypertensive emergencies.^1^ Hypertensive urgencies are defined as severe elevations in blood pressure (diastolic blood pressure above 120 mmHg) without evidence of acute, progressive target organ damage.^2^^,^^3^^,^^4^^,^^5^ The target organs are primarily the heart, brain, kidneys and large arteries. Hypertensive emergencies consist of elevated blood pressure (BP) with evidence of target organ dysfunction and have been the subject of a separate Cochrane review.^6^

Hypertension is common and affects about 50 million individuals in the United States and approximately one billion people worldwide.^7^ In Brazil, cardiovascular diseases are responsible for more than 250,000 deaths annually.^7^ Hypertensive urgencies are important clinical events occurring in both hospital and outpatient settings and comprise about 76% of hypertensive crises.^8^

In these situations, patients should be carefully evaluated with detailed history-taking and physical examination.^9^ The need for treatment is considered urgent but allows for slow control using oral or sublingual drugs.^9^ As chronic hypertension results in a shift in cerebrovascular autoregulation, in which blood pressure decreases too rapidly, to below the lower limit of autoregulation, the brain may become hypoperfused, with symptoms such as dizziness, nausea and syncope.^10^ For this reason, if the increase in blood pressure is not associated with risk to life or acute target-organ damage, blood pressure control must be implemented slowly over 24 hours.^9^ Excessively rapid reductions in BP have been associated with acute deterioration in renal function and ischemic cardiac or cerebral events.^11^

Most patients with severe BP elevation can be managed on an outpatient basis with oral agents and appropriate follow-up within 24 hours to several days, depending on the individual characteristics of the patient.^9^ The initial goal for BP reduction is not to attain normal blood pressure but, rather, to achieve a progressive, controlled reduction in BP in order to minimize the risk of hypoperfusion in the cerebral, coronary and renovascular regions.^10^

For hypertensive urgencies, it is not known which class of antihypertensive drug provides the best results in terms of morbidity, mortality, blood pressure lowering efficacy, withdrawal due to adverse effects and other side effects. There is controversy regarding when to use blood pressure drugs and which ones to use in these situations, and the available evidence has been insufficient to answer these questions.

## OBJECTIVE

To assess the effectiveness and safety of oral drugs for hypertensive urgencies.

## MATERIALS AND METHODS

This systematic review of the literature was developed in accordance with the methodology of the Cochrane Collaboration and was conducted at the Brazilian Cochrane Centre, in the Universidade Federal de São Paulo - Escola Paulista de Medicina (Unifesp-EPM). It was approved by the local ethics committee.

The review only included randomized controlled clinical trials that evaluated the use of one or more drugs in the calcium channel blocker (CCB) or angiotensin-converting enzyme inhibitor (ACEi) groups. The participants in the trials had to meet all the criteria below:

- Diastolic blood pressure elevation to more than 110 mmHg and no evidence of acute target-organ damage.^4^ A lower pressure than in the current definition was used because we did not want to lose studies and because, prior to 1993, many cases of hypertensive urgencies were defined and treated with diastolic blood pressures ≥ 115 mmHg or ≥ 110 mmHg.
- Age over 18 years.
- Patients with pregnancy-related and eclampsia-related hypertension were excluded.
- Patients with intractable nosebleed, sympathomimetic drug overdose, hypertension associated with increased circulating catecholamines, end-stage organ damage (hypertensive emergencies), or other conditions requiring parenteral therapy were excluded.^5^

The outcomes evaluated were total mortality (from cardiovascular causes, from any cause or from side effects of the medication); any adverse effects reported in the studies included; proportion of patients with blood pressure decrease (for four-hour and 24-hour periods); proportion of patients with target blood pressure of 140/90 mmHg; decrease in blood pressure in mmHg (for systolic blood and diastolic blood pressure); number of patients requiring addition of a second or third drug; incidence of hospitalization due to any cause; total non-fatal cerebrovascular, cardiovascular and cardiopulmonary events (stroke, myocardial infarction, angina, silent ischemia, arrhythmias, congestive heart failure, kidney failure and acute pulmonary edema); and time taken to achieve target BP.

### Search strategy for identifying studies

The search strategy included the following databases: Medical Literature Analysis and Retrieval System Online (Medline) [1996 to January 2007]; Cochrane Systematic Review Database; Literatura Latino-Americana e do Caribe em Ciências da Saúde (Lilacs) [1996 to January 2007]; Excerpta Medica Database (Embase) [1996 to January 2007]; and specific websites (http://www.controlledtrials.com, http://clinicaltrials.gov/ct/gui, http://www.CenterWatch.com, http://scielo.br). Pharmaceutical industry representatives, specialists in the field and the main authors of the trials included were contacted to obtain access to unpublished data. There were no language restrictions. The terms used in the databases are available in Table 1.

**Table 1.** Search strategies

Database	Search strategy
Medline	#1 (“Nifedipine”[Mesh]) OR (Procardia XL) OR (Adalat) OR (Bay-1040) OR (BAY-a-1040) OR (Cordipin) OR (Cordipine) OR (Corinfar) OR (Korinfar) OR (Fenigidin) OR (Infedipin) OR (Nifangin) OR (Nifedipine Monohydrochloride) OR (Monohydrochloride, Nifedipine) OR (Nifedipine-GTIS) OR (Procardia) OR (nifedipine) #2 (“Captopril”[MeSH]) OR ((S)-1-(3-Mercapto-2-methyl-1-oxopropyl)-L-proline)) OR (Capoten) OR (Lopirin) OR (SQ-14,225) OR (SQ 14,225) OR (SQ14,225) OR (SQ-14225) OR (SQ 14225) OR (SQ14225) OR (SQ-14,534) OR (SQ 14,534) OR (SQ14,534) OR (SQ-14534)OR (SQ 14534) OR (SQ14534) #3 (“Calcium Channel Blockers”[Mesh]) OR (Exogenous Calcium Antagonists) OR (Antagonists, Exogenous Calcium) OR (Calcium Antagonists, Exogenous) OR (Exogenous Calcium Blockaders) OR (Blockaders, Exogenous Calcium) OR (Calcium Inhibitors, Exogenous) OR (Calcium Channel Blocking Drugs) OR (Exogenous Calcium Inhibitors) OR (Inhibitors, Exogenous Calcium) OR (Calcium Blockaders, Exogenous) OR (Channel Blockers, Calcium) OR (Blockers, Calcium Channel) OR (Calcium Channel Blocker) #4 (“Angiotensin-Converting Enzyme Inhibitors”[Mesh]) OR (Angiotensin Converting Enzyme Inhibitors)OR (Angiotensin-Converting Enzyme Antagonists) OR (Angiotensin Converting Enzyme Antagonists) OR (Enzyme Antagonists, Angiotensin-Converting) OR (Antagonists, Angiotensin-Converting Enzyme) OR (Antagonists, Angiotensin Converting Enzyme) OR (Antagonists, Kininase II) OR (Inhibitors, Kininase II) OR (Inhibitors, ACE) OR (ACE Inhibitors) OR (Kininase II Inhibitors) OR (Kininase II Antagonists) OR (Angiotensin I-Converting Enzyme Inhibitors) OR (Angiotensin I Converting Enzyme Inhibitors) OR (Inhibitors, Angiotensin-Converting Enzyme) OR (Enzyme Inhibitors, Angiotensin-Converting) OR (Inhibitors, Angiotensin Converting Enzyme) #5 (hypertensive urgenc*) OR (“Hypertensive Encephalopathy”[Mesh]) OR (“Hypertension/complications” [MeSH]) OR (severe AND hypertension) OR (hypertensive AND crisis) OR (acute AND hypertens*) OR (acute AND treatment AND hypertension) OR (acute AND blood AND pressure AND lowering AND effect) OR (malignant AND hypertension) OR (accelerat* AND hypertension) OR (hypertensive AND encephalopat*) #6 #1 OR #2 OR #3 OR #4 AND #5 AND (randomized controlled trial [pt] OR controlled clinical trial [pt] OR randomized controlled trials [mh] OR random allocation [mh] OR double-blind method [mh] OR single-blind method [mh] OR clinical trial [pt] OR clinical trials [mh] OR (“clinical trial” [tw]) OR ((singl* [tw] OR doubl* [tw] OR trebl* [tw] OR tripl* [tw]) AND (mask* [tw] OR blind* [tw])) OR ( placebos [mh] OR placebo* [tw] OR random* [tw] OR research design [mh:noexp] OR comparative study [mh] OR evaluation studies [mh] OR follow-up studies [mh] OR prospective studies [mh] OR control* [tw] OR prospectiv* [tw] OR volunteer* [tw]) NOT (animals [mh] NOT humans [mh])
Cochrane Database	#1 nifedipine #2 captopril #3 calcium channel blocker$ #4 angiotensin converting enzyme inhibitor$ #5 hypertensive urgenc$
Lilacs	#1 (Nifedipine) or (Procardia XL) or (Adalat) or (Bay-1040) or (BAY-a-1040) or (Cordipin) or (Cordipine) or (Corinfar) or (Korinfar) or (Fenigidin) or (Infedipin) or (Nifangin) or (Nifedipine Monohydrochloride) or (Monohydrochloride, Nifedipine) or (Nifedipine-GTIS) or (Procardia) or (nifedipine) #2 (Captopril) or ((S)-1-(3-Mercapto-2-methyl-1-oxopropyl)-L-proline)) or (Capoten) or (Lopirin) or (SQ-14,225) or (SQ 14,225) or (SQ14,225) or (SQ-14225) or (SQ 14225) or (SQ14225) or (SQ-14,534) or (SQ 14,534) or (SQ14,534) or (SQ-14534) or (SQ 14534) or (SQ14534) #3 (Calcium Channel Blockers) OR (Exogenous Calcium Antagonists) OR (Antagonists, Exogenous Calcium) OR (Calcium Antagonists, Exogenous) OR (Exogenous Calcium Blockaders) OR (Blockaders, Exogenous Calcium) OR (Calcium Inhibitors, Exogenous) OR (Calcium Channel Blocking Drugs) OR (Exogenous Calcium Inhibitors) OR (Inhibitors, Exogenous Calcium) OR (Calcium Blockaders, Exogenous) OR (Channel Blockers, Calcium) OR (Blockers, Calcium Channel) OR (Calcium Channel Blocker) #4 (Angiotensin-Converting Enzyme Inhibitors) or (Angiotensin Converting Enzyme Inhibitors) or (Angiotensin-Converting Enzyme Antagonists) or (Angiotensin Converting Enzyme Antagonists) or (Enzyme Antagonists, Angiotensin-Converting) or (Antagonists, Angiotensin-Converting Enzyme) or (Antagonists, Angiotensin Converting Enzyme) or (Antagonists, Kininase II) or (Inhibitors, Kininase II) or (Inhibitors, ACE) or (ACE Inhibitors) or (Kininase II Inhibitors) or (Kininase II Antagonists) or (Angiotensin I-Converting Enzyme Inhibitors) or (Angiotensin I Converting Enzyme Inhibitors) or (Inhibitors, Angiotensin-Converting Enzyme) or (Enzyme Inhibitors, Angiotensin-Converting) or (Inhibitors, Angiotensin Converting Enzyme) #5 #1 OR #2 OR #3 OR #4 #6 (hypertensive urgenc$) or (Hypertensive Encephalopathy) or (Hypertension/complications) OR (severe AND hypertension) OR (hypertensive AND crisis) OR (acute AND hypertens$) OR (acute AND treatment AND hypertension) OR (acute AND blood AND pressure AND lowering AND effect) OR (malignant AND hypertension) OR (accelerat$ AND hypertension) OR (hypertensive AND encephalopat$) #7 ((Pt ENSAIO CONTROLADO ALEATORIO OR Pt ENSAIO CLINICO CONTROLADO OR Mh ENSAIOS CONTROLADOS ALEATORIOS OR Mh DISTRIBUICAO ALEATORIA OR Mh MÉTODO DUPLO-CEGO OR Mh MÉTODO SIMPLES-CEGO or PT ESTUDO MULTICENTRICO) or ((tw ensaio or tw ensayo or tw trial) and (tw azar or tw acaso or tw placebo or tw control$ or tw aleat$ or tw random$ or (tw duplo and tw cego) or (tw doble and tw ciego) or (tw double and tw blind)) and tw clinic$)) AND NOT ((Ct ANIMAIS OR ct coelhos or ct camundongos or MH ANIMAIS OR MH RATOS OR MH PRIMATAS OR MH CAES OR MH COELHOS OR MH SUINOS) AND NOT (Ct HUMANO AND Ct ANIMAIS)) #8 #5 AND #6 AND #7
Embase	1 ‘hypertensive crisis’/exp AND [humans]/lim 2 ‘hypertensive urgency’ AND [humans]/lim 3 #1 OR #2 4 ‘angiotensin receptor antagonist’/exp AND [humans]/lim 5 ‘captopril’/exp AND [humans]/lim 6 #4 OR #5 7 ‘calcium channel blocking agent’/exp AND [humans]/lim 8 ‘nifedipine’/exp AND [humans]/lim 9 #7 OR #8 10 #3 AND (#6 OR #7 OR #8) AND [humans]/lim

Medline = Medical Literature Analysis and Retrieval System Online; Lilacs = Literatura Latino-Americana e do Caribe em Ciências da Saúde; Embase = Excerpta Medica Database; MeSH = Medical Subject Headings.

### Data extraction and methodological quality assessment

The search strategy identified the relevant articles. Each of these articles was assessed by two independent reviewers. All the data were extracted by these two reviewers. Details relating to the population, treatment periods and demographic baseline were extracted independently. A third reviewer was consulted to help in resolving disagreements. The quality of each trial was evaluated independently by the two reviewers, using the validated quality assessment tool that was published by Jadad et al. in 1996.^12^

### Statistical analysis and presentation of the results

The statistical analysis was carried out using the ReviewManager program (version 5.0, RevMan, 2000), and in accordance with the Cochrane Collaboration Handbook.^13^ For dichotomous variables, the odds ratio (OR) method was used, with 95% confidence intervals (random effect model). When there was a statistical difference, the number needed to treat (NNT) or the number needed to harm (NNH) was calculated. For continuous variables, the weighted mean difference was calculated (random effect model) with the corresponding 95% confidence interval. If necessary, the original data were transformed into a logarithmic basis to obtain better distribution, or into scales that presented similar properties (the data on this scale would be the input for meta-analysis). Furthermore, if necessary, the continuous variables were subdivided for dichotomous analysis. To analyze the sensitivity, the following strategy using the Review Manager 5.0 software^14^ was proposed:

- Reanalysis of the data using reasonable variation of values for lost data: when dichotomous variables were extracted, it was assumed that participants lost from the experimental group presented unsuccessful treatment and that losses from the control group presented improvement;
- Reanalysis of the data using reasonable variation of the results from the studies, when there was some uncertainty in the results;
- Reanalysis of the data using different statistical methods;
- Statistical heterogeneity: it was planned that this would be evaluated in the studies by inspection of the graphical presentation (a dispersion graph in which the study weight or sample size was put on the y-axis, versus the risk ratio on the x-axis), and by the heterogeneity test (chi-squared test with n degrees of freedom, in which n was the number of studies that contributed data, minus one).^13^

## RESULTS

Sixteen randomized controlled trials^15^^,^^16^^,^^17^^,^^18^^,^^19^^,^^20^^,^^21^^,^^22^^,^^23^^,^^24^^,^^25^^,^^26^^,^^27^^,^^28^^,^^29^^,^^30^ (769 patients) met the inclusion criteria for this review. We excluded 58 clinical trials for several reasons:

- One randomized controlled trial included the same patients as in a previous study.^31^
- Twelve trials mixed patients with and without acute target-organ damage in the same randomized controlled trial.^32^^,^^33^^,^^34^^,^^35^^,^^36^^,^^37^^,^^38^^,^^39^^,^^40^^,^^41^^,^^42^^,^^43^
- Eighteen trials included non-randomized participants in the trial results.^44^^,^^45^^,^^46^^,^^47^^,^^48^^,^^49^^,^^50^^,^^51^^,^^52^^,^^53^^,^^54^^,^^55^^,^^56^^,^^57^^,^^58^^,^^59^^,^^60^^,^^61^
- Five trials had inadequate randomization.^62^^,^^63^^,^^64^^,^^65^^,^^66^
- One trial did not report any of the outcomes of interest.^67^
- Five trials did not fulfill the blood pressure threshold criteria.^68^^,^^69^^,^^70^^,^^71^
- Two trials did not fulfill the patient threshold criteria.^72^^,^^73^
- One had a double-dummy design.^74^
- Thirteen trials compared interventions that were not within the scope of this review.^75^^,^^76^^,^^77^^,^^78^^,^^79^^,^^80^^,^^81^^,^^83^^,^^84^^,^^85^^,^^86^

For the purposes of statistical analysis, the comparisons were made according to the outcomes, by comparing the following groups: 1) CCB versus ACEi; 2) placebo versus CCB; 3) placebo versus ACEi; 4) CCB versus other interventions; and 4) ACEi versus other interventions.

### Outcomes

Total mortality: No trial reported total mortality.

**Figure 1.**
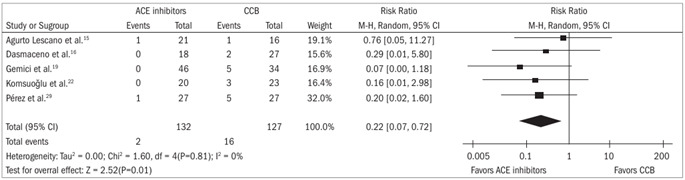
Forest plot for comparison between angiotensin-converting enzyme inhibitors and calcium channel blockers, in relation to the outcome of flushing.

**Figure 2.**
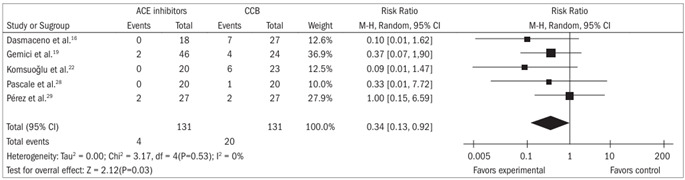
Forest plot for comparison between angiotensin-converting enzyme inhibitors and calcium channel blockers, in relation to the outcome of headache

Adverse effects: There were significant differences favoring participants receiving ACEi drugs, compared with CCB drugs, concerning adverse effects such as flushing^15^^,^^16^^,^^19^^,^^22^^,^^29^ (risk ratio 0.22; 95% confidence interval 0.07 to 0.72) and headache^16^^,^^19^^,^^22^^,^^28^^,^^29^ (risk ratio 0.34; 95% confidence interval 0.13 to 0.92) (Figures 1 and 2).

Proportion of patients with blood pressure decrease: Only one study described this outcome as part of the results,^30^ and it showed that the proportion of blood pressure decrease over four hours was 92% for both groups (captopril and nifedipine).

Proportion of patients with target blood pressure of 140/90 mmHg: No trial reported this outcome.

**Figure 3.**
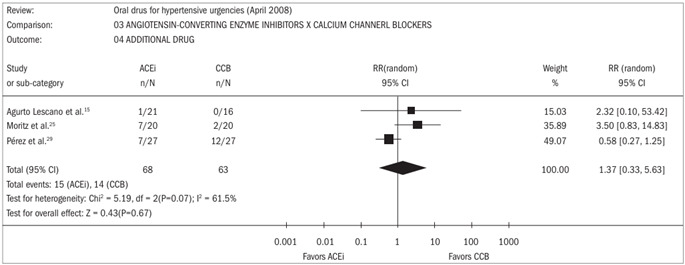
Forest plot for comparison between angiotensin-converting enzyme inhibitors and calcium channel blockers, in relation to the outcome of number of patients requiring addition of a second or third drug.

Number of patients requiring addition of a second or third drug: Four trials^15^^,^^24^^,^^25^^,^^29^ reported this outcome. Two patients in the CCB group and four patients in the control group (other drugs) required administration of an additional drug.^24^ There were no significant differences favoring participants receiving ACEi drugs compared with CCB drugs, in relation to this outcome (Figure 3).^15^^,^^25^^,^^29^

Incidence of hospitalization due to any cause: One trial^29^ reported that one patient needed hospitalization, and two cases of hospitalization: one case due to treatment failure with CCB use and the other case due to treatment failure with ACEi drugs.

Total non-fatal cerebrovascular, cardiovascular and cardiopulmonary events: One trial^19^ reported one patient with angina after taking CCB to treat hypertensive urgency.

Time taken to achieve target blood pressure: This was reported in 10 trials.^15^^,^^16^^,^^17^^,^^22^^,^^23^^,^^24^^,^^26^^,^^28^^,^^29^^,^^30^ The results relating to this outcome were very variable, and meta-analysis could not be performed because of the absence of data to calculate the standard deviation and lack of definition of the desired target blood pressure. For the CCB group, the time needed for blood pressure reduction ranged from 30 minutes^30^ to 100 minutes.^15^ For the ACEi group, this time ranged from 30 minutes^30^ to 120 minutes.^15^^,^^16^

According to the Jadad Scale, the quality assessment was as follows: three trials received one point (described as randomized, inadequate randomization, no double-blinding, no withdrawals description),^17^^,^^27^^,^^28^ four trials received two points (described as randomized, adequate randomization, no double-blinding and no withdrawals description),^16^^,^^25^^,^^29^^,^^30^ six trials received three points (described as randomized, adequate randomization, described as double-blinded, inadequate double-blinding and no withdrawals description)^18^^,^^19^^,^^20^^,^^22^^,^^23^^,^^29^ and three trials received four points (described as adequate randomization, adequate double-blinding, no withdrawals description).^15^^,^^21^^,^^26^

## DISCUSSION

This was the first systematic review investigating mortality and morbidity outcomes among all randomized controlled trials (RCT) on drug treatments for hypertensive urgencies. The Cochrane Collaboration methodology was followed closely by conducting extensive literature search, followed by critical evaluation of RCT found.

A previous systematic review that combined hypertensive emergencies and urgencies did not include 11 trials that were included in our systematic review and, furthermore, it mixed randomized with non-randomized trials.^3^ The hypertensive urgencies included in that review had been treated with a variety of agents, and the main drugs used were captopril (a type of ACEi) and nifedipine (a type of CCB). Perez’s review investigated mortality and morbidity outcomes among all randomized controlled trials on drug treatment for hypertensive emergencies.^6^

The studies included in the present review had many limitations. First, there were large variations and inconsistencies in the definitions and cutoffs for urgencies and emergencies and for target blood pressures. In 13 of the 58 trials excluded, patients with hypertensive urgencies and emergencies were mixed or were not clearly discriminated in the same trial.^75^^,^^76^^,^^77^^,^^78^^,^^79^^,^^80^^,^^81^^,^^83^^,^^84^^,^^85^^,^^86^ If it had been possible to obtain the data on the individual patients, the ones with hypertensive urgencies could have been added to our review. Second, there was a lack of definition regarding urgencies and short-term trials. Third, important clinical outcomes were often not measured. Finally, the small numbers of patients (an average of 48 patients per trial) in the studies included limited their power to detect differences in mortality and morbidity.

## CONCLUSIONS

### Implications for practice

Evidence currently exists to suggest that the use of oral ACEi drugs for hypertensive urgencies produces better outcomes with regard to effectiveness and lower frequency of adverse effects, compared with CCB drugs. Thus, when possible, oral ACEi drugs should be used, except during pregnancy.

### Implications for research

Randomized controlled trials are needed to assess different blood pressure lowering strategies and different drug classes in patients with hypertensive urgencies. The outcomes measured in such trials should be the following: total mortality; any adverse effects reported; blood pressure reduction (proportion of patients with blood pressure decrease for four-hour and 24-hour periods, and proportion of patients with target blood pressure of 140/90 mmHg); systolic and diastolic blood pressure decrease (in mmHg); time taken to achieve target blood pressure; number of patients requiring additional drugs; incidence of hospitalization due to any cause; and total non-fatal cerebrovascular, cardiovascular and cardiopulmonary events, including at least 24 hours of monitoring follow-up for all patients.

We believe that further collaborative, multicenter, randomized double-blind controlled trials need to be performed in order to answer these questions more appropriately.
